# Apelin Counteracts the Effects of *Fusobacterium nucleatum* on the Migration of Periodontal Ligament Cells In Vitro

**DOI:** 10.3390/ijms251910729

**Published:** 2024-10-05

**Authors:** Pablo Cores Ziskoven, Andressa V. B. Nogueira, Sigrun Eick, James Deschner

**Affiliations:** 1 Department of Periodontology and Operative Dentistry, University Medical Center of the Johannes Gutenberg University, 55131 Mainz, Germany; pablo.cores@unimedizin-mainz.de (P.C.Z.); a.nogueira@uni-mainz.de (A.V.B.N.); 2 Department of Periodontology, School of Dental Medicine, University of Bern, 3010 Bern, Switzerland; sigrun.eick@unibe.ch

**Keywords:** apelin, periodontal ligament cells, *Fusobacterium nucleatum*, periodontitis, obesity

## Abstract

To better understand the link between periodontitis and metabolic diseases, our in vitro study aimed to assess the influence of the adipokine apelin and/or the periodontal pathogen *Fusobacterium nucleatum* on periodontal cells. Periodontal ligament (PDL) cells were exposed to *F. nucleatum* in the presence and absence of apelin. Scratch assays were used to analyze the in vitro wound healing and velocity of cell migration. To investigate if *F. nucleatum* and/or apelin have a regulatory effect on cell proliferation and apoptosis, proliferation and viability assays were performed as well as an analysis of caspase 9 expression. Both the in vitro wound closure and the cell migration rate were significantly reduced by *F. nucleatum.* Simultaneous incubation with apelin counteracted the adverse effects of *F. nucleatum*. The proliferation assay demonstrated that neither apelin nor *F. nucleatum* significantly affected PDL cell proliferation. Furthermore, neither apelin nor *F. nucleatum* was cytotoxic or affected apoptosis after 48 h. Apelin could play a modulatory role in the pathogenesis of periodontitis, as it was able to compensate for the inhibitory effects of the periodontal pathogen *F. nucleatum* on PDL cell migration in vitro.

## 1. Introduction

Periodontitis is a chronic inflammatory disease of the tooth-supporting tissues, which is associated with irreversible soft and hard tissue degradation and resorption, which can lead to increased tooth mobility and even tooth loss [1]. Due to various local or systemic risk factors, e.g., smoking, immunosuppression, medication and stress, a homeostasis breakdown can occur in the symbiotic biofilm on the tooth surface, causing the biofilm to become dysbiotic. Although the entire biofilm is, amongst other factors, responsible for the development of periodontitis, keystone pathogens, e.g., *Porphyromonas gingivalis*, are of particular importance for the development of dysbiosis. The biofilm dysbiosis is also associated with a breakdown of the balance between the periodontal microorganisms and the host response [2,3]. As mentioned above, numerous risk factors play a role in the initiation and progression of periodontitis [4,5,6,7,8,9,10,11,12,13,14,15,16]. In addition to genetic and epigenetic predisposition [17,18,19,20,21], there is strong evidence that periodontal diseases are also causally associated with systemic conditions or diseases [22,23], such as cardiovascular disease [24], rheumatoid arthritis [25], type II diabetes mellitus (T2DM) [26], obesity and metabolic syndrome [27].

Individuals suffering from overweight or obesity may exhibit dysregulated production of cytokines by cells of the adipose tissue [28]. These so-called adipokines are messenger substances, which are of physiological and pathophysiological significance and exert a variety of effects on different cell types and tissues [29,30]. Among other activities, they are capable of regulating thirst and hunger sensations, insulin metabolism, angiogenesis, bone metabolism, energy balance, coagulation, hematopoiesis, as well as inflammation and its suppression [31,32,33]. Adipose tissue is therefore not regarded as a pure energy store but as an active metabolic organ whose secretion of adipokines is dysregulated in the case of excessive adipose tissue [28].

Various studies have shown that adipokines are also produced in oral cells, that their synthesis is regulated by numerous local and systemic factors, and that such adipokines influence many functions of periodontal cells. Adiponectin has anti-inflammatory effects by counteracting proinflammatory and proteolytic molecules, whereas resistin, visfatin, and leptin exert proinflammatory actions, e.g., by increasing the gene expression of matrix metalloproteinase (MMP)-1, C-C motif chemokine ligand (CCL) 2 and tumor necrosis factor (TNF)-α [29,34,35,36,37,38,39,40]. In contrast, little is known about the role of the more recently described adipokine apelin in the etiopathogenesis of periodontitis and its link to metabolic diseases. In one study, the serum levels of apelin in periodontally and systemically healthy individuals and in periodontitis patients with and without T2DM were analyzed [41]. The study showed that apelin concentrations were significantly higher in periodontitis patients than in the healthy control group. Furthermore, apelin concentrations were highest in patients suffering from both periodontitis and T2DM, suggesting that apelin may play a role in periodontitis and glucose regulation. Similar results were also found in saliva samples by other investigators who examined the apelin concentration in the saliva of periodontally diseased diabetics as well as periodontally and systemically healthy individuals [42]. Again, the highest apelin concentrations were found in patients with periodontitis and T2DM. The increased systemic apelin concentrations in diabetes and obesity could represent a compensatory mechanism for the reduced insulin sensitivity in T2DM.

Our research group has recently demonstrated for the first time that apelin concentrations are not only altered systemically in serum and saliva but also locally in gingival crevicular fluid samples [43]. In a recent in vitro study, we also showed that apelin and its receptor are locally expressed in the periodontium and that apelin is able to enhance the periodontopathogen *Fusobacterium nucleatum*-induced increase in the expression of proinflammatory cytokines, chemokines, and proteases [44]. Apelin may therefore contribute to periodontal inflammation and tissue destruction [45]. The aim of the present in vitro study was to further explore the pathomechanistic links between periodontitis and metabolic diseases by investigating possible effects of apelin on the actions of *F. nucleatum* with respect to wound closure, migration, proliferation and viability of PDL cells. The null hypothesis of this study was that apelin has no effect on *F. nucleatum*-regulated in vitro wound healing.

## 2. Results

### 2.1. Effects of F. nucleatum and/or Apelin on In Vitro Wound Closure and Velocity of Cell Migration

**Figure 1:**
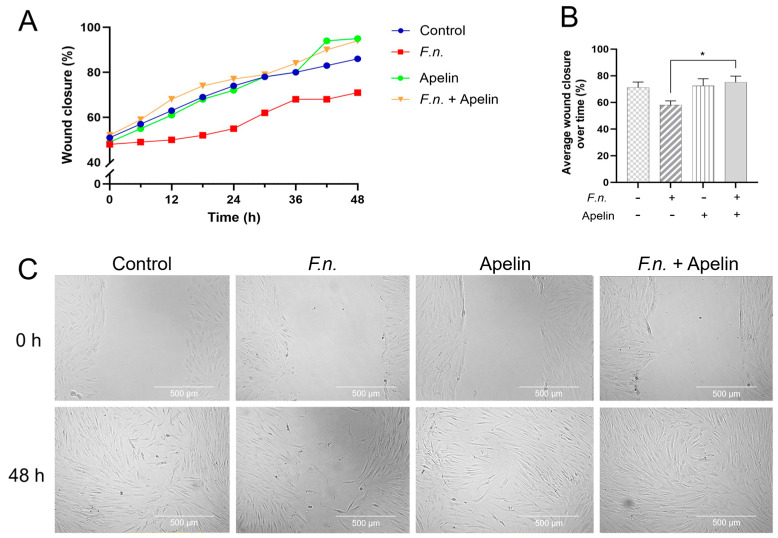
(**A**) In vitro wound closure of the cell-free area (%) by PDL cells under different conditions (blue: control; red: *F. nucleatum* (OD_660_: 0.025); green: apelin (1 ng/ml); yellow: *F. nucleatum* + apelin) over 48 h. (**B**) Average in vitro wound closure of the cell-free area (%) by PDL cells exposed to *F. nucleatum* (OD_660_: 0.025) and/or apelin (1 ng/ml), as shown in (A). A minus sign indicates the absence of *F. nucleatum* or apelin, whereas a plus sign indicates the presence of *F. nucleatum* or apelin. (**C**) Representative light microscope images of in vitro wound closure of PDL cell monolayers in the presence and absence of *F. nucleatum* (OD_660_: 0.025) and/or apelin (1 ng/ml) at 0 h and 48 h. Untreated cells served as control. * significantly (*p* < 0.05) different.

**Figure 2:**
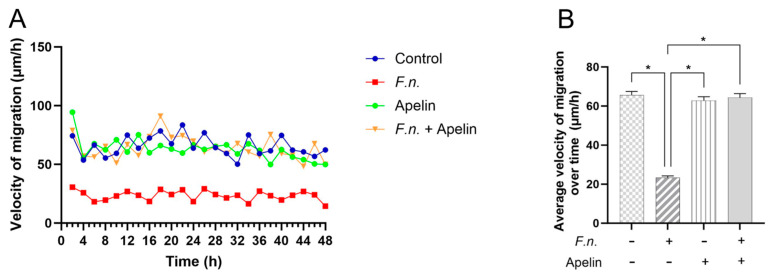
(**A**) Velocity of PDL cell migration (μm/h) under different conditions (blue: control; red: *F. nucleatum* (OD_660_: 0.025); green: apelin (1 ng/ml); yellow: *F. nucleatum* + apelin) over 48 h. (**B**) Average migration velocity (μm/h) of PDL cells exposed to *F. nucleatum* (OD_660_: 0.025) and/or apelin (1 ng/ml), as shown in (**A**). A minus sign indicates the absence of *F. nucleatum* or apelin, whereas a plus sign indicates the presence of *F. nucleatum* or apelin. Untreated cells served as control. * significantly (*p* < 0.05) different.

After incubation of injured PDL cell monolayers with *F. nucleatum* and/or apelin, wound closure was analyzed over 48 h. As shown in Figure 1A–C, after 48 h, the percentage of cell-free area was highest in the monolayers incubated with *F. nucleatum* alone, i.e., *F. nucleatum* inhibited wound closure. Apelin alone had no effect on the wound healing. When the *F. nucleatum*-incubated monolayers were simultaneously treated with apelin, the wound closure was significantly (*p* < 0.05) improved compared with the monolayers treated with *F. nucleatum* alone, i.e., apelin was able to significantly (*p* < 0.05) antagonize the inhibitory effect of *F. nucleatum*. The mean migration velocity was significantly (*p* < 0.05) lower in the monolayers incubated with *F. nucleatum* alone than in all other groups (Figure 2A,B). The cells of the control, apelin and combined *F. nucleatum* and apelin groups migrated at a similar average velocity.

### 2.2. Effects of F. nucleatum and/or Apelin on Cell Proliferation

**Figure 3:**
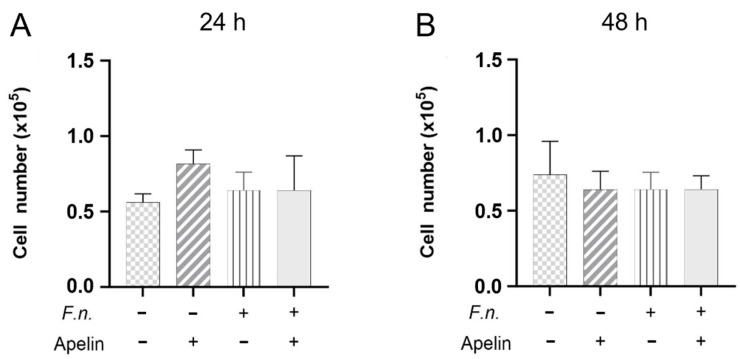
Analysis of the average number of PDL cells in vitro in the presence and absence of *F. nucleatum* (OD_660_: 0.025) and/or apelin (1 ng/ml) at 24 h (**A**) and 48 h (**B**). A minus sign indicates the absence of *F. nucleatum* or apelin, whereas a plus sign indicates the presence of *F. nucleatum* or apelin. Untreated cells served as control.

Since the wound closure also depends on the cell number, the effects of *F. nucleatum* and/or apelin on the number of cells were evaluated. As shown in Figure 3A,B, *F. nucleatum* and/or apelin had no significant effects on the PDL cell numbers after 24 h and 48 h.

### 2.3. Effects of F. nucleatum and/or Apelin on Cell Viability and Apoptosis

**Figure 4:**
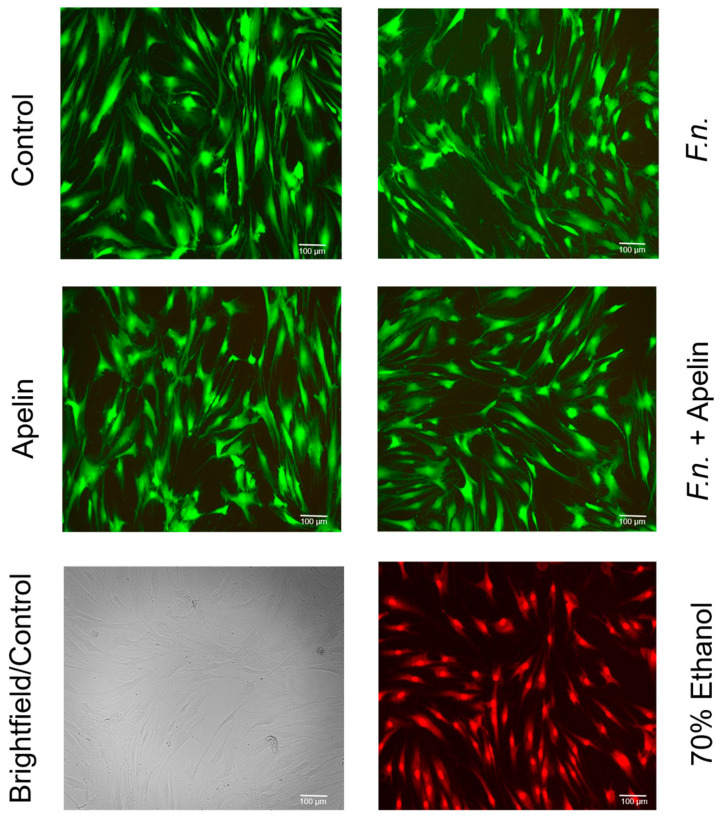
Viability of PDL cells in the presence and absence of *F. nucleatum* (OD_660_: 0.025) and/or apelin (1 ng/ml) in vitro at 48 h, as analyzed by live and dead assay and light microscopy. Unfiltered image (brightfield) and positive control for dead cells (70 % ethanol).

**Figure 5:**
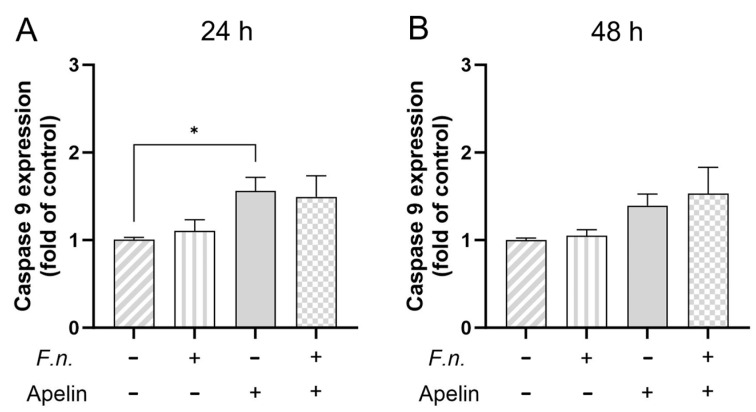
Effects of *F. nucleatum* (OD_660_: 0.025) and/or apelin (1 ng/ml) on the expression of caspase 9 at 24 h (**A**) and 48 h (**B**). A minus sign indicates the absence of *F. nucleatum* or apelin, whereas a plus sign indicates the presence of *F. nucleatum* or apelin. Untreated cells served as control. * significant (*p* < 0.05) difference between groups.

Live and dead assay was used to test whether *F. nucleatum* and/or apelin could affect viability. Images of merged “green” and “red” channels are shown (Figure 4). Almost no red-labeled cells were found (with the exception of the positive control for dead cells), indicating that the cells remained viable. In addition, cell morphology was also not affected by *F. nucleatum* and/or apelin. Figure 5A,B shows the relative gene expression of caspase 9 in PDL cells treated with *F. nucleatum* and/or apelin after 24 h and 48 h. While no significant regulation by *F. nucleatum* and/or apelin was observed after 48 h, a slight but significant increase in caspase-9 gene expression after apelin incubation was noticed after 24 h.

## 3. Discussion

The experiments of this study showed that the oral pathogen *F. nucleatum* was able to significantly inhibit the in vitro wound healing and the velocity of PDL cell migration after 48 h. Interestingly, the adipokine apelin counteracted the inhibitory effect of *F. nucleatum* on both the wound healing and cell migration. Our in vitro experiments therefore suggest that apelin may play a modulatory role in the pathogenesis of periodontitis.

Interestingly, the effects of *F. nucleatum* and/or apelin on wound closure were not mediated through changes in cell number. Moreover, the further analyses also revealed that *F. nucleatum* and/or apelin had no significant effects on cell viability and apoptosis after 48 h. Additionally, no changes in cell morphology after incubation of the cells with *F. nucleatum* and/or apelin were observed. Only the velocity of cell migration was influenced by *F. nucleatum*, and this inhibitory effect of *F. nucleatum* on cell migration was antagonized by apelin. This may suggest that the protective effect of apelin on wound healing observed in this study was achieved through the stimulatory impact of this adipokine on cell migration.

Gingival and periodontal healing proceeds like any wound healing in the body with the overlapping phases of inflammation/exudation (first hours), resorption (1–4 days), granulation/proliferation (3–10 days) and the longer lasting repair phase (7 days to months) [46]. Migration, proliferation and viability of fibroblasts are crucial in the initial and later phases of tissue healing in the oral cavity [47]. It is known from the literature that obesity is associated with reduced wound healing capacity for various reasons [48,49,50]. The relationship between periodontitis and obesity has also been extensively studied and is well described [51]. Adipokines, such as apelin, may provide a pathomechanistic explanation for the negative impact of metabolic diseases on periodontal tissues [29]. For this reason, in our study, periodontal fibroblasts were exposed to apelin in the presence and absence of *F. nucleatum*. Inflammation-promoting effects of other adipokines such as leptin, visfatin and resistin on periodontal cells and tissues have already been described in vitro by our research group [36,37,39]. Apelin, whose serum and salivary concentrations are elevated in patients with diabetes and/or periodontitis, is a relatively understudied adipokine in the periodontium [41,42]. Recently, we have found that not only are systemic apelin concentrations altered in the context of periodontitis, but that different levels may also occur at the local level in the gingival sulcus [43].

It is well known that healing processes can be positively or negatively influenced [52,53,54,55,56,57]. Therefore, an in vitro wound healing assay was performed to determine possible effects of *F. nucleatum* and/or apelin on PDL cells in vitro. The experiments of the present study showed that *F. nucleatum* exerts inhibitory effects on wound closure and PDL cell migration, which could be abrogated by apelin. This opposing effect of *F. nucleatum* and apelin is in contrast to our recent observations where apelin further enhanced the actions of *F. nucleatum* [44]. In these experiments, apelin led to a further increase in *F. nucleatum*-induced expression of proinflammatory cytokines such as COX2, CCL2, CXCL8 and TNF-α. Such ambivalent, i.e., partly tissue-promoting and partly tissue-destroying, effects were also observed in vitro when investigating the adipokine leptin [39]. In this study, the effects of leptin and/or enamel matrix derivative (EMD) on the regenerative capacity of PDL cells were investigated using a scratch assay and real-time PCR. Leptin had negative effects on markers of hard and soft tissue remodeling, but was able to significantly enhance the EMD-induced promoting effects on cell migration of PDL cells. The present study revealed no significant effects of *F. nucleatum* and/or apelin on proliferation, viability and apoptosis, at least for the time points examined, which is consistent with the results of other studies [58]. Interestingly, our previous in vitro experiments have shown that apelin was able to increase the *F. nucleatum*-stimulated expression and synthesis of proteases [44]. Although these results suggest a destructive component, this effect could facilitate the migration of the cells and, thus, the in vitro wound closure. This mechanism, known as “focal proteolysis” [59], creates a pathway with less mechanical stress so that the cells follow this gradient. Since proteases can therefore promote cell migration, it is conceivable that apelin could possibly modulate the wound healing and cell migration in the presence of *F. nucleatum* by regulating such proteolytic molecules. As shown in our previous study, *F. nucleatum* in combination with apelin led to a significantly increased gene expression of the chemokine CCL2, which could also be confirmed at the protein level [44]. The increased levels of this chemokine induced by apelin and *F. nucleatum* could possibly also explain the improved migration observed in this study in the presence of apelin, as CCL2 is a strong promoter of cell migration in different cell types [60,61,62].

Monolayer cell culture models have been used in a variety of studies with different cells and for a wide range of questions to investigate wound healing and migration in vitro [63]. The in vitro scratch model is a relatively simple model to construct and has the great advantage that it includes numerous different cell functions (e.g., proliferation, viability, migration, etc.) and reflects the overall effect as the wound closure rate. Using this in vitro simulation of wound healing, in vivo experiments, which are frequently associated with ethical, time and financial disadvantages, can often be avoided. Like any in vitro model, this model can only assess a small part of wound healing and only under limited conditions and a short time frame. Wound healing in vivo is of course much more complex because different cell types are involved, vascularization plays an important role, etc. Future studies on animal models should show whether our results from the in vitro model can also be reproduced in a complex situation. As mentioned above, our in vitro study has limitations. It has already been pointed out that in vitro wound healing can only simulate some aspects of complex healing. Furthermore, an investigation of in vitro wound healing with this model is only possible over a few days, as at least one group finally achieved complete wound closure. Our additional experiments and analyses regarding proliferation, viability and gene expression were therefore also based on the duration of our in vitro wound healing model. Future studies with other models should analyze whether the effects observed here also occur with longer study durations.

In the present study, we investigated the regulatory effects of *F. nucleatum* and/or apelin on caspase 9 as a marker of apoptosis. Numerous molecules are involved in the regulation of apoptosis. However, since caspase 9 is upstream of the apoptosis cascade, this molecule is of central importance in the regulation of programmed cell death [64]. Nevertheless, future studies should also examine other markers of apoptosis in regard to the activities of *F. nucleatum* and apelin. It would also be interesting to know whether the enzyme activity of caspase-9 differs from the expression data in our study.

Furthermore, we focused on PDL cells in this study. The investigation of other periodontal cells, such as gingival keratinocytes and fibroblasts or bone cells, would contribute to a more comprehensive view of complex periodontal healing. Finally, clinical studies in gingivitis and periodontitis patients should investigate the role of apelin in etiopathogenesis and therapy.

The apelin concentration used in the present study has already been applied by other researchers [65,66]. This concentration was also used in our earlier in vitro experiments [44]. This makes it possible to compare our results with those of other studies and our own. Unfortunately, there are still very few studies that could provide a clue as to the actual concentrations of apelin found in oral tissues and fluids under different physiological and pathophysiological conditions. In a recent clinical study conducted by our research group, we were able to identify concentrations in the gingival crevicular fluid that were within the range used in the present study [43]. Nonetheless, the exact concentrations within the PDL are unknown and may even vary between different sites.

Among the periodontopathogenic microorganisms, we focused on *F. nucleatum*, as in our previous studies, because it is a bridging microorganism that plays an important role in the development and progression of gingivitis and periodontitis, and for reasons of comparability with our previous studies. Furthermore, *F. nucleatum* is one of the most frequently detected species in oral biofilms, emphasizing its importance in health and disease [67,68]. However, it should be emphasized that periodontitis is a multispecies disease. Therefore, the study of other microorganisms and their combinations would be of further benefit in the future. As in our previous protocols, we used *F. nucleatum* as a lysate, which most likely contained LPS, but also other virulence factors such as proteases, which may have been involved in the effects of *F. nucleatum* in our experiments.

Future studies should also address the mechanisms by which apelin is able to counteract *F. nucleatum*-inhibited wound healing. Some studies have shown that apelin can affect the cytoskeleton of fibroblasts. For example, triggering the apelin signaling pathway leads to increased cell migration and synthesis of migration-associated molecules such as actin and vimentin [69]. In another study on epithelial cells, it was also shown that apelin promotes the synthesis of cytoskeletal proteins [70]. Moreover, apelin activated the expression of proteins of the PI-3K/Akt and MAPK/Erk signaling pathways, such as PLCγ1, p38, Akt and Erk phosphorylation in these cells. Interestingly, *F. nucleatum* has been shown to activate the PI3K-AKT/MAPK/NF-κB signaling pathways in macrophages [71]. Furthermore, the *F. nucleatum* adhesin FadA could activate the Raf1-MAPK and IKK-NF-κB signaling pathways in PDL cells [72]. Earlier studies by our group also demonstrated that *F. nucleatum* activates the MAPK signaling pathway in periodontal fibroblasts [44,73]. Finally, there is also evidence that *F. nucleatum* activates the AKT/MAPK and NF-κB signaling pathways in gingival fibroblasts [74]. Overall, these studies suggest that apelin and *F. nucleatum* regulate the same intracellular signaling pathways involved in cell migration and wound healing and may therefore influence each other’s effects.

In conclusion, both cell migration and wound closure were significantly reduced by *F. nucleatum* in vitro, and apelin counteracted the adverse effects of *F. nucleatum*. The null hypothesis that apelin has no effect on *F. nucleatum*-regulated in vitro wound healing was thus rejected. Our in vitro experiments, therefore, suggest that apelin may play a modulating role in the pathogenesis of periodontitis.

## 4. Materials and Methods

### 4.1. Cell Culture

A human PDL cell line was used for cell culture [75]. The cell line was obtained from the third molar of a healthy 26-year-old male non-smoker after obtaining written consent according to the ethics regulations of the University of Goettingen (file no.: 27/2/09). Immortalization with human telomerase reverse transcriptase was performed, which has been described before [76]. Cells were cultured in cell culture flasks provided with nutrient medium. The cell culture medium was Dulbecco’s Modified Eagle Medium (DMEM) GlutaMAX (Thermo Fisher Scientific, Waltham, MA, USA) supplemented with 10% fetal bovine serum (FBS, Thermo Fisher Scientific), 100 units of penicillin and 100 μg/ml streptomycin (Thermo Fisher Scientific). Cells were maintained in the incubator at 37 °C and with a humidified atmosphere of 5% CO_2_. Cells were cultivated (1 × 10^5^ cells/well) on 6-well culture plates and grown until 70–80% confluence. Medium change was performed every other day, and the FBS concentration was reduced to 1% 24 h before cell treatment. The oral pathogenic bacterium *F. nucleatum* ATCC 25586 was used at an optical density (OD_660nm_) of 0.025 in order to simulate microbial infection in vitro. Bacterial strain was pre-cultivated on Schaedler agar plates (Oxoid, Basingstoke, UK) in an anaerobic atmosphere for 48 h. Next, bacteria were suspended in phosphate-buffered saline (OD_660nm_ = 1, corresponding to 1.2 × 10^9^ bacterial cells/ml) and submitted twice to ultrasonication (160 W for 15 min) leading to complete bacterial killing. Moreover, apelin (recombinant human apelin protein, Abcam, Cambridge, United Kingdom) was used for in vitro stimulation in the concentration of 1 ng/ml, as previously reported [44,65,66]. Untreated cells served as control.

### 4.2. In Vitro Wound Healing Assay and Cell Migration

To analyze the in vitro wound healing and cell migration, PDL cells were grown on 35 mm plastic culture dishes (Greiner Bio-One, Frickenhausen, Germany) until 100% confluence. A 200 µl pipette tip (Starlab International, Hamburg, Germany) was used to scratch a reproducibly 3–4 mm-wide cell-free area, which represents the wound. Then, all non-adherent cells were removed by washing steps with cell culture medium. In the course of the experiment, the manner in which this area was again overgrown by cells until the wound closed could be observed. The cells were additionally incubated with *F. nucleatum* (OD_660nm_: 0.025) and/or apelin (1 ng/ml). Untreated cells served as a control group. In order to assess the wound closure rate by the scratch assay, the cells had to be photographed reproducibly at different time points. For this purpose, the JuLi™ BR (NanoEnTek, Seoul, South Korea) cell movie analyzer was used. This device consists of two chambers, which are equipped with microscope and camera. Cells were analyzed throughout 48 h. For analysis, one image was used every 6 h (time points: 6 h, 12 h, 18 h, 24 h, 30 h, 36 h, 42 h and 48 h). To evaluate the photographs, the open-source program ImageJ (version: 1.54g) was used [77]. The plugin “MRI_Wound_Healing_Tool” [78] was added to this program to automatically detect the cell-free areas. The images were calibrated using a scale. The same sections of the image were always used without zooming in or out.

Another approach we followed to quantify migration speed was by tracking single cells using ImageJ and the plugin “Manual_Tracking” (2005, Fabrice Cordelires, Institut Curie, Orsay, France). For this purpose, the complete image stacks of an experiment were imported into the ImageJ program and the position in the first image was marked. In the course of the experiment, it was possible to scroll through the stack and target the previously marked cell again in each subsequent image. The plugin thus keeps track of the migration path. Since the image was calibrated using a scale, the speed and distance traveled between each image could be measured. The program calculates the background and displays the distance as points and lines. Six cells from each group were marked as examples from the three tests. Mean values were formed for comparison. The experiment was repeated three times.

### 4.3. Analysis of Cell Number

To analyze the number of PDL cells under different conditions, cells were grown on 6-well culture plates according to the protocol described previously. At 70–80% confluence, the medium was reduced to 1% FBS and cells were exposed to *F. nucleatum* (OD_660nm_: 0.025) and/or apelin (1 ng/mL). After 24 h and 48 h, cells were counted. For this purpose, cell trypsinization was performed and after resuspending the cells in medium, 100 µl of the cell suspension was combined with 100 µl of trypan blue solution (Thermo Fisher Scientific). In the following step, 10 µl of the mixture was loaded to LUNA™ Cell Counting Slides (Logos Biosystems, Anyang, South Korea) at both sides. The slide was then inserted into the cell counter device (LUNA-II^TM^ Automated Cell Counter, Logos Biosystems), and the number of cells per ml per sample was registered.

### 4.4. Analysis of Cell Viability

To verify whether *F. nucleatum* and/or apelin have a cytotoxic effect or affect apoptosis, the LIVE/DEAD Viability/Cytotoxicity Kit (L3224, Molecular Probes, Thermo Fisher Scientific) was used. This kit can be used to determine the live cell content of cell populations by fluorescence microscopy. The principle is based on the esterase activity found in living cells. These enzymes can cleave calcein to calcein-AM, which fluoresces green at a wavelength of λ = ~495/~515 nm. Dead cells can, in turn, be detected using ethidium homodimer (EthD-1). This molecule infiltrates cells whose cell walls are defective and thus marks dead cells. At a wavelength of λ = ~495/~635 nm, it fluoresces red. EthD-1 is emitted from living cells. For the present experiment, cells were grown on 24-well plates at a density of 20,000 cells per well according to the protocol described. At 70–80% confluence, FBS was reduced to 1%, and the cells were treated on the following day according to the experimental design for 24 h and 48 h. For fluorescence microscopy, the staining solutions (2 μM Calcein and 4 μM EthD-1) were mixed with 10 ml sterile PBS by vortexing. After the cells were washed twice with PBS, 500 μl of the staining solution was added. Fluorescence microscopy was performed by using ZOE™ (Fluorescent Cell Imager, Bio-Rad Laboratories, Munich, Germany). Untreated cells served as control. The experiment was arranged in triplets and repeated three times.

### 4.5. Real-Time PCR

RNA extraction was performed using the RNeasy Mini Kit (Qiagen, Hilden, Germany) according to the manufacturer’s instructions. NanoDrop ND-2000 (Thermo Fischer Scientific) spectrophotometer was used in order to determine the RNA concentration. Five hundred ng of total RNA was reverse-transcribed using the iScrip Select cDNA Synthesis Kit (Bio-Rad Laboratories, Munich, Germany) according to the manufacturer’s protocol. Gene expression analysis of glyceraldehyde-3-phosphate dehydrogenase (GAPDH) and caspase 9 was performed using the PCR thermal cycler CFX96 (Bio-Rad Laboratories), SYBR green PCR master mix (QuantiFast SYBR Green PCR Kit, Qiagen), and specific primers (QuantiTect Primer Assay, Qiagen). One µl of cDNA was mixed with 12.5 µl master mix, 2.5 µl primer and 9 µl nuclease-free water. The mix was heated at 95 °C for 5 min, followed by 40 cycles of denaturation at 95 °C for 10 s and a combined annealing/extension step at 60 °C for 30 s. Data were analyzed by the comparative threshold cycle method.

### 4.6. Statistical Analysis

The statistical analysis was performed using the software GraphPad Prism (GraphPad 10.2.3 Software, San Diego, CA, USA). For data analysis, mean values and standard errors of the mean (SEM) were calculated. Data were checked for normal distribution and subsequently analyzed with the *t*-test (parametric) or Mann–Whitney-U test (non-parametric). For multiple comparisons, ANOVA or the Kruskall–Wallis test was applied, depending on normal distribution. The Dunnet’s (parametric) or Dunn’s test (non-parametric) served as post hoc tests. The significance level was set at *p* < 0.05 for all experiments.

## Data Availability

The datasets presented in this article are not readily available because of ongoing studies. Requests to access the datasets should be directed to the corresponding author.
